# Lights On or Off? The Efficacy of Light Therapy as Add‐On to Cognitive Behavioural Therapy for Insomnia—A Pilot Study

**DOI:** 10.1111/jsr.70357

**Published:** 2026-06-04

**Authors:** Sarah R. Schmid, Christine Blume, Johanna Ell, Nikolas Konzen, Matthias Apweiler, Bernd Feige, Bernd L. Fiebich, Julian E. Schiel, Katharina Domschke, Kai Spiegelhalder

**Affiliations:** ^1^ Department of Psychiatry and Psychotherapy, Medical Center—University of Freiburg, Faculty of Medicine University of Freiburg Freiburg Germany; ^2^ Centre for Chronobiology Psychiatric Hospital of the University of Basel Basel Switzerland; ^3^ Research Cluster Molecular and Cognitive Neurosciences University of Basel Basel Switzerland; ^4^ Department of Biomedicine University of Basel Basel Switzerland; ^5^ Neuroimmunology and Neurochemistry Research Group, Department of Psychiatry and Psychotherapy, Medical Center—University of Freiburg, Faculty of Medicine University of Freiburg Freiburg Germany; ^6^ German Center for Mental Health (DZPG) Partner Site Berlin/Potsdam Berlin Germany

**Keywords:** CBT‐I, insomnia, light therapy, sleepiness

## Abstract

Cognitive behavioural therapy for insomnia (CBT‐I) is recommended as first‐line treatment for insomnia disorder. However, around 25% of the patients do not respond to the treatment, and only 40% achieve full remission. The current study investigates the therapeutic potential of light therapy as an add‐on to CBT‐I. Insomnia severity and questionnaire‐related secondary outcomes were assessed at baseline, post‐treatment and follow‐up. Sleep diary‐related variables and the cortisol awakening response were assessed at pre‐ and post‐treatment. Linear mixed models were calculated for each outcome variable. The study included 42 patients with insomnia disorder. Reduction in insomnia severity did not significantly differ between groups from baseline to post‐treatment (*β* = 0.35, SE = 1.31, *p* = 0.792). Regarding exploratory secondary outcomes, daytime sleepiness was significantly more reduced from baseline to follow‐up in the CBT‐I + light therapy group than in the CBT‐I + placebo light group (*β* = −1.05, SE = 0.43, *p* = 0.020). There was no significant condition × time point interaction effect for any of the other secondary outcomes. The findings show no significant add‐on effect of light therapy on insomnia severity. However, light therapy had a significant effect on daytime sleepiness that became evident only at the follow‐up. Given that daytime functioning is of high clinical relevance for patients with insomnia disorder, daytime sleepiness should be further evaluated as treatment target and may be addressed with light therapy.

**Trial Registration:**

ClinicalTrials.gov identifier: DRKS00029640

## 1 Introduction

Insomnia disorder, that is, difficulties with sleep initiation or maintenance associated with at least one form of daytime impairment, is among the most prevalent mental disorders (Morin et al. 2015). Approximately 10% of the individuals in Western industrialised nations fulfil the diagnostic criteria for insomnia disorder, with women being at higher risk for difficulties with initiating (OR = 2.26) or maintaining (OR = 2.05) sleep as well as for early morning awakening (OR = 1.49) compared to men (Kocevska et al. 2021). Insomnia disorder is associated with cognitive impairments (Wardle‐Pinkston et al. 2019) and physical complaints (e.g., headaches or gastrointestinal issues) (Kyle et al. 2010; Léger and Bayon 2010). Furthermore, insomnia increases the risk for the development of other mental disorders, especially depression and anxiety disorders (Hertenstein et al. 2019; Dressle et al. 2022), as well as for somatic diseases (Gibson et al. 2023). Insomnia disorder also has a significant impact on health economic costs. The annual economic burden of insomnia has been estimated at approximately $100 billion in the United States (Léger and Bayon 2010). Taken together, insomnia disorder is a significant risk factor for the most prevalent mental disorders and physical diseases and results in a high economic burden.

Cognitive behavioural therapy for insomnia (CBT‐I) is recommended as first‐line treatment for insomnia disorder by current clinical guidelines due to its high efficacy (Morin et al. 2024; Riemann et al. 2023; van Straten et al. 2018). Meta‐analytic results show that the majority of patients benefit significantly from CBT‐I; however, around 25% of the patients do not respond to the treatment and full remission is only achieved in around 40% of the patients (Morin et al. 2009). Thus, there remains the need to extend CBT‐I. One potential approach is to enhance treatment outcomes through the addition of adjunctive therapies. A promising but yet understudied tool may be the therapeutic application of light. Light functions as the most important zeitgeber for the human internal biological clock that in turn synchronises the sleep–wake cycle with the light–dark cycle (Aschoff et al. 1969). In addition, light has an enhancing effect on alertness (Aemmi et al. 2020) and mood and is successfully applied in seasonal affective disorders. Preliminary meta‐analytical findings show reduced insomnia symptoms after morning administration of light therapy as standalone treatment (van Maanen et al. 2016; Chambe et al. 2023). However, it remains unclear whether light therapy has an additional benefit when administered together with CBT‐I, and the potential additive effect is the focus of the present investigation.

Insomnia is associated with depressive symptoms (Hertenstein et al. 2019) and moderately increased cortisol levels (Dressle et al. 2022). Interestingly, light therapy is recommended as a treatment of seasonal affective disorder by current guidelines (Bundesärztekammer (BÄK) et al. 2022). In addition, morning light administration has an impact on the cortisol awakening response in healthy subjects (i.e., the strong increase in cortisol levels within 30–45 min after morning awakening [Stalder et al. 2025]), indicating a light‐dependent impact on the circadian rhythm. Hence, the present study also aimed at investigating the effects of light therapy on depressive symptoms and the cortisol awakening response.

Taken together, the current randomised placebo‐controlled trial aimed at investigating the efficacy of light therapy as an add‐on to CBT‐I in patients with insomnia disorder. The primary hypothesis was that CBT‐I + light therapy has a stronger effect on post‐treatment insomnia severity than CBT‐I + placebo light. Secondary outcomes included depressive symptoms, the cortisol awakening response and other sleep‐related variables.

## 2 Methods

### 2.1 Participants

Between October 2022 and December 2024, 42 patients were included in this study. All of them (1) fulfilled the diagnostic criteria for insomnia disorder according to the DSM‐5, (2) were at least 18 years old and (3) provided written informed consent. Individuals were excluded when they met one or more of the following exclusion criteria: (1) evidence of other sleep disorders according to the International Classification of Sleep Disorders (ICSD‐3); (2) psychoactive medication or sleep medication (i.e., benzodiazepines, benzodiazepine receptor agonists, sedating antidepressants, orexin receptor antagonists, antipsychotics, melatonin and antihistamines, taken specifically to improve sleep) intake 2 weeks prior or during the study period; (3) shift work or time zone flight within the 4 weeks before or during the study period; (4) history of treatment with CBT‐I or light therapy; (5) acute or poorly managed chronic pain; (6) substance abuse or substance use disorder; (7) evidence of intellectual disability; (8) participation in other clinical trials during the last 4 weeks before study participation; (9) evidence of physical conditions precluding treatment with light therapy and (10) unstable medical conditions.

### 2.2 Procedure

The study flow of the current trial is presented in Figure 1. Participants were recruited via the internal hospital website of the Medical Center—University of Freiburg. Interested individuals were screened via telephone interview (30–60 min) conducted by a clinical psychologist specialist in sleep medicine or by a master's student in psychology under the supervision of the clinical psychologist. The semi‐structured interview comprised questions about sleep history, other sleep disorders and physical diseases, as well as the Mini‐DIPS—a standardised screening instrument based on DSM‐5 diagnostic criteria for mental disorders (Margraf et al. 2017). In the next step, participants were invited to the clinic for a physical examination and routine blood work (blood cell count, renal, liver and thyroid function), carried out by a physician or a medical student under the supervision of the physician. Included patients completed the baseline assessment (T0) comprising a 1‐week sleep diary and questionnaires about insomnia severity (Insomnia Severity Index [ISI] Bastien et al. 2001), depressive symptoms (Beck Depression Inventory‐II [BDI‐II] Beck et al. 1996), daytime sleepiness (Epworth‐Sleepiness Scale [ESS] Johns 1991), sleep quality (Pittsburgh Sleep Quality Index [PSQI] Buysse et al. 1998) and chronotype (Composite Scale of Morningness [CSM] Randler 2014). To assess morning cortisol levels, the participants were asked to collect three saliva samples at home at baseline. After the baseline assessment, participants were randomised in blocks of two to either the CBT‐I + light therapy or the CBT‐I + placebo light condition. Randomisation was carried out by a scientist otherwise not involved in the current trial. All patients received a face‐to‐face treatment with CBT‐I (see Section 2.4). Group allocation was concealed from the therapists, and participants were instructed not to reveal details about the lamp in order to maintain double blinding. Participants were informed only that the study involved two different light conditions with distinct characteristics. No further details about the specific nature of the light conditions were provided. In case of technical problems with the therapy lamps, the participants were advised to contact technical staff. The post‐treatment (T1) assessment was carried out as soon as possible after the last CBT‐I session and comprised the same measures as at T0, except for the CSM. In addition, participants again collected three saliva samples at home to assess morning cortisol levels.

**FIGURE 1:**
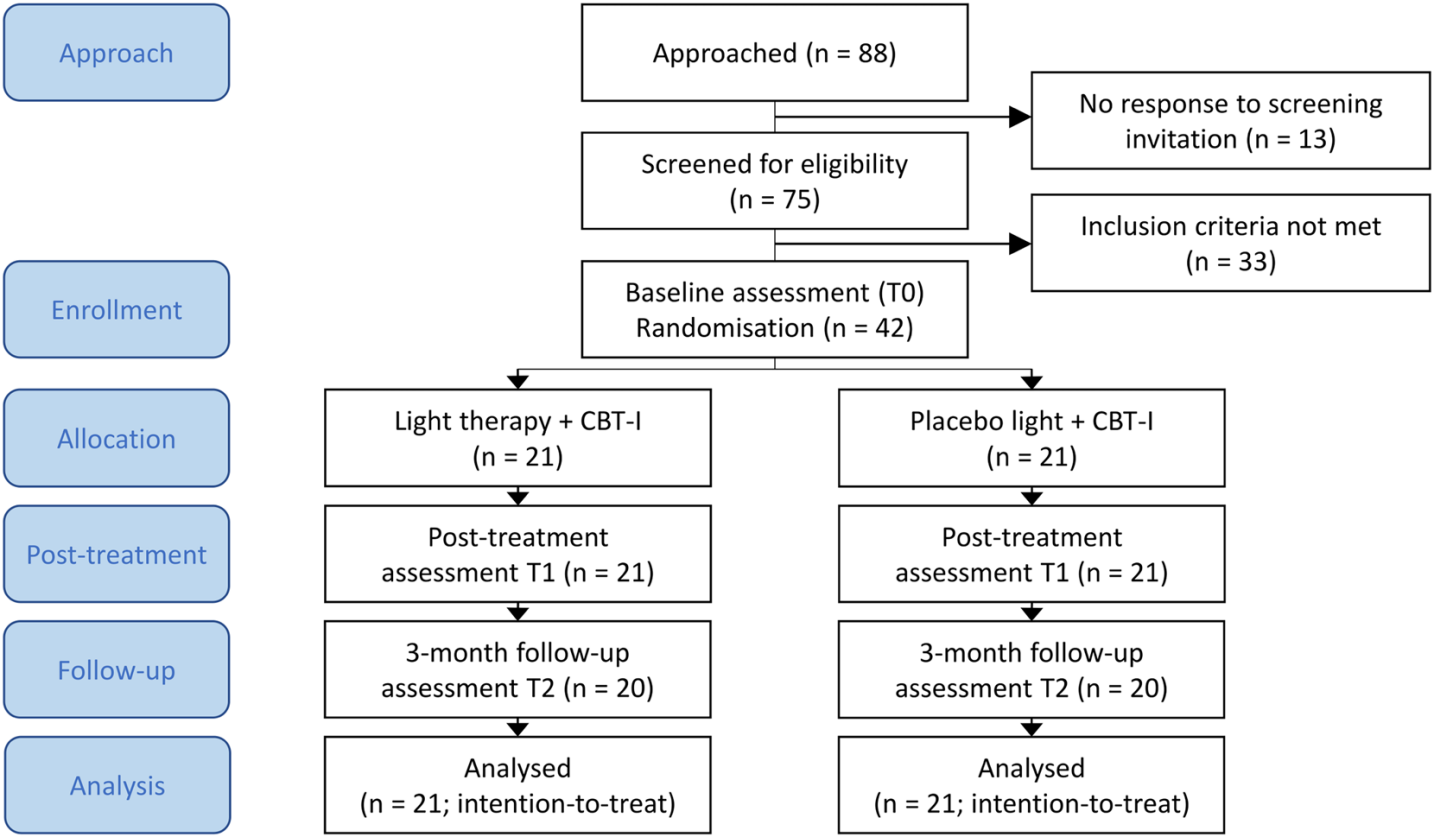
Flow chart of the randomised controlled trial.

The study was conducted in accordance with the Declaration of Helsinki and was registered in the German Clinical Trial Register (DRKS00029640). The local ethics committee of the Medical Center—University of Freiburg approved the study protocol (application no. EK‐Freiburg: 21‐1641).

### 2.3 Interventions

Both the light therapy and the placebo light therapy were applied by the participants at home using modified daylight lamps (HALO; Lumie, Cambridge, UK). The participants were asked to use their lamp within the first 2 h after awakening, at least four times a week for a period of 20 min at a distance of 20 cm. The duration was chosen based on feasibility considerations. Participants were instructed to look directly into the lamp without wearing contact lenses or glasses and to record their lamp usage times. At the time of lamp distribution, the setup was demonstrated once in person without turning on the lamp in order to maintain blinding. The lamps for the two conditions differed in illuminance level and colour temperature. According to the manufacturer's specifications, the light therapy lamp emitted an illuminance of 12,480 photopic lux, a colour temperature of 3687 K and 7356 lx melanopic EDI; the placebo lamp emitted 60 photopic lux, a colour temperature of 2949 K and 28 lx melanopic EDI (both measured at a distance of 20 cm at eye level; light values were calculated using luox.app Spitschan et al. 2022).

CBT‐I was applied in a face‐to‐face setting at the Medical Center—University of Freiburg, by a clinical psychologist specialised in sleep medicine. The treatment consisted of four weekly individual treatment sessions of 50 min each. Manual‐based CBT‐I included psychoeducation, relaxation therapy, sleep restriction therapy, stimulus control therapy and cognitive therapy (Spiegelhalder et al. 2011). Sleep restriction and stimulus control were introduced in the second therapy session and typically began on the same day. During sleep restriction therapy, the initial sleep window was set based on the average sleep duration of the preceding 2 weeks according to sleep diary data. The timing of the sleep window was chosen by patient preference, and the minimum time in bed was 4.5 h. On a weekly basis, time in bed was increased by 30 min when the average sleep efficiency of the preceding seven nights was > 90%, decreased by 30 min when sleep efficiency was < 80% and maintained when sleep efficiency was between 80% and 90%. Stimulus control therapy was performed following Bootzin (1972). Progressive muscle relaxation was used as a relaxation intervention, and cognitive therapy included cognitive restructuring and the ‘worry chair’ technique.

### 2.4 Saliva Sampling and Laboratory Analyses

Saliva samples were collected using Salivettes (Sarstedt, Nümbrecht, Germany) by the patients at home in the morning directly after awakening, 30 and 60 min after awakening, following the recommendation by Stalder and colleagues (Stalder et al. 2016) of three post‐awakening sampling time points in adults. Patients were advised that they should not eat, brush teeth or drink anything other than water until after the last sampling. Salivettes were stored after collection at −18°C at the participants' home for a maximum of 48 h until they were stored at the clinical laboratory at −80°C. Salivettes were centrifuged at 1000 × *g* for 2 min at room temperature to collect the samples, and cortisol was measured using Cortisol Saliva ELISA kits (EUROIMMUN Medizinische Labordiagnostika AG, Lübeck, Germany) strictly following the manufacturer's protocol. Briefly, 50 μL of the appropriate standards, controls and samples were transferred onto the pre‐coated microplate and 100 μL peroxidase‐labelled cortisol were added to all wells. After 90 min of incubation at room temperature, the plate was washed and 100 μL of chromogen/substrate solution was added and incubated for another 15 min. In the next step, 100 μL of stop solution was added and the photometric reaction was determined using an MRX^e^ Microplate reader (Dynex Technologies, Denkerdorf, Germany) with a wavelength of 450 nm and a reference wavelength of 630 nm. The cortisol awakening response was operationalised as the area under the curve (AUC) with respect to the increase (AUCi), a well‐established measure estimating state‐dependent changes over time (Pruessner et al. 2003).

### 2.5 Statistical Analyses

For descriptive purposes, means and standard deviations were calculated for continuous variables and proportions for categorical variables. Efficacy analyses were conducted following intention‐to‐treat principles, and missing data at T1 and T2 were imputed using the last‐observation‐carried‐forward method. Linear mixed models were used in order to analyse pre‐ to post‐treatment effects with the independent variables ‘condition’ (light therapy vs. placebo) and ‘time point’ (T1 vs. T0) and the ‘condition × time point’ interaction as well as ‘study ID’ as a random effect. In order to test for effects becoming evident only at follow‐up (Dekker et al. 2020), linear‐mixed models with the independent variables ‘condition’ (light therapy vs. placebo) and ‘time point’ (T2 vs. T0) and the ‘condition × time point’ interaction, as well as ‘study ID’ as a random effect, were used. Of note, one patient in the placebo condition did not complete the questionnaires at T0 and was therefore excluded from the corresponding analyses. For sleep diary data, mean values were calculated for each variable for the pre‐treatment week and the post‐treatment week. If data were not available for the full week, only the available data were used. The primary outcome was the ISI score at T1. Treatment response (ISI change > 6 from T0 to T1) and remission (ISI score < 8 at T1) were reported descriptively (Morin et al. 2009). Exploratory secondary outcomes included (1) the following questionnaire scores: BDI‐II score, ESS score and PSQI score, (2) the following sleep diary‐derived variables: sleep efficiency (SE), total sleep time (TST), wake after sleep onset (WASO) and sleep onset latency (SOL), as well as (3) the cortisol awakening response. All statistical analyses were conducted using the statistical software R, version 4.3.2. Statistical analyses were performed with the base R package; processing of the cortisol data was performed using the R package dplyr (Wickham et al. 2023). The level of significance was set at < 0.05 (two‐tailed). A power analysis using G*Power (version 3.1.9.7; Faul et al. 2007) indicated that a total sample size of 42 participants (21 per group) yielded a statistical power (1 − *β*) of 89% to detect a medium effect size (*f* = 0.25) for the within‐between interaction in the primary analysis, assuming an alpha level of 0.05 and a correlation of *r* = 0.5 among repeated measures.

## 3 Results

### 3.1 Study Population

The study sample included 42 patients with insomnia disorder. The mean age at T0 was 51.7 ± 16.9 years (CBT‐I + light therapy condition: 52.7 ± 16.9 years; CBT‐I + placebo condition: 50.7 ± 17.3 years, *t* = 3.88, *p* = 0.700); 30 patients (71.4%) were female (16 in the CBT‐I + light therapy condition and 14 in the CBT‐I + placebo light condition, *χ* ^2^ = 0.12, *p* = 0.733) and the mean ISI score was 15.9 ± 3.4 at baseline (light therapy condition: 15.5 ± 3.8; placebo condition: 16.4 ± 3.1, t = 0.82, *p* = 0.418). Regarding comorbidities, three participants met the criteria for an anxiety disorder (one in the CBT‐I + light therapy condition and two in the CBT‐I + placebo light condition) and one participant in the light therapy condition had a history of recurrent depressive disorder, currently in remission. In both groups, 21 patients completed the T1 assessment and 20 patients the T2 assessment. Further information about the sample is presented in Table 1.

**TABLE 1:** Effects on questionnaires and self‐reported sleep.

Outcome	Groups						Pre‐ to post‐comparisons between groups			Pre to follow‐up comparisons between groups		
	CBT‐I + light therapy			CBT‐I + placebo light			Condition × time point			Condition × time point		
	T0	T1	T2	T0	T1	T2	*β*	SE	*p*	*β*	SE	*p*
ISI	15.5 (3.8)	9.5 (4.1)	8.6 (4.5)	16.4 (3.1)	10.1 (3.1)	11.3 (4.4)	0.35	1.31	0.792	−0.90	0.87	0.307
BDI‐II	11.9 (8.2)	6.6 (6.4)	7.5 (7.8)	13.1 (8.7)	7.9 (7.9)	7.8 (7.2)	−0.13	1.79	0.941	0.41	1.09	0.708
ESS	7.7 (4.0)	6.8 (4.6)	4.9 (2.6)	8.0 (3.9)	7.1 (3.4)	7.3 (3.1)	0.00	1.09	1.000	−1.05	0.43	0.020
PSQI	8.9 (3.4)	6.1 (2.8)	6.2 (2.9)	10.5 (3.3)	6.3 (2.4)	7.7 (3.4)	1.39	1.10	0.213	0.04	0.57	0.941
CSM	36.7 (7.4)	—	—	35.2 (6.2)	—	—	—	—	—	—	—	—
SE	0.74 (0.13)	0.87 (0.05)	—	0.72 (0.10)	0.84 (0.09)	—	0.01	0.04	0.799	—	—	—
TST	373.0 (72.6)	371.0 (58.5)	—	360.2 (65.7)	374.9 (47.5)	—	−18.76	13.49	0.172	—	—	—
WASO	51.2 (38.2)	19.9 (13.2)	—	49.0 (42.8)	22.0 (25.0)	—	−6.90	10.81	0.530	—	—	—
SOL	41.1 (42.5)	13.5 (10.6)	—	39.1 (22.6)	24.8 (18.6)	—	−15.20	9.14	0.104	—	—	—
Bedtime	22:59 (40.8)	23:25 (34.5)	—	23:26 (48.5)	23:31 (36.7)	—	20.99	14.43	0.153	—	—	—
Awakening time	06:49 (65.8)	06:11 (56.0)	—	07:05 (67.2)	06:38 (61.1)	—	−17.41	12.89	0.185	—	—	—
Rise time	07:26 (72.9)	06:24 (56.0)	—	07:46 (56.9)	07:02 (51.1)	—	−12.35	13.06	0.350	—	—	—
Midsleep point	03:14 (52.8)	02:55 (39.2)	—	03:34 (55.3)	03:18 (45.8)		−8.40	10.61	0.433	—	—	—
AUCi	271.8 (96.8)	317.1 (92.1)	—	339.3 (77.9)	317.1 (92.1)	—	75.81	105.96	0.619	—	—	—

*Note:* Descriptive data by condition and time point are reported as mean (standard deviation).

Abbreviations: AUCi, area under the curve with respect to the cortisol increase; BDI‐II, Beck Depression Inventory‐II; CSM, Composite Scale of Morningness; ESS, Epworth Sleepiness Scale; ISI, Insomnia Severity Index; PSQI, Pittsburgh Sleep Quality Index; SE, sleep efficiency; SOL, sleep onset latency; TST, total sleep time; WASO, wake after sleep onset.

### 3.2 Primary Outcome

Concerning the ISI change from T0 to T1, the interaction effect condition × time point was not significant (*β* = 0.35, SE = 1.31, *p* = 0.792), indicating that the CBT‐I + light therapy condition did not elicit a significantly greater decrease from T0 to T1 (15.5–9.5) compared with the CBT‐I + placebo light condition (16.4–10.1). The result did not substantially change when controlling for BDI‐II scores at T0 (*β* = 0.38, SE = 1.22, *p* = 0.764). In the CBT‐I + light therapy condition, 10 patients (47.6%) were categorised as responders and six patients (28.6%) as remitters at T1. In the CBT‐I + placebo light condition, seven patients (33.3%) were categorised as responders and only two (9.5%) as remitters at T1, indicating no significant differences between groups regarding responders (*χ* ^2^ = 0.25, *p* = 0.615) and remitters (*χ* ^2^ = 1.22, *p* = 0.269). At T2, 13 patients in the CBT‐I + light therapy condition were categorised as responders and nine patients were categorised as remitters. In the CBT‐I + placebo light condition, eight patients were categorised as responders and three as remitters, indicating no significant between‐group differences in either remission rate (*χ* ^2^ = 2.61, *p* = 0.106) or response rate (*χ* ^2^ = 1.19, *p* = 0.276).

### 3.3 Secondary Outcomes

Results for all questionnaire‐ and sleep diary‐based outcomes are presented in Table 1. On average, participants completed sleep diary entries on 6.7 days. Of these entries, 3.3% were missing for TST, 13.1% for WASO and 5.6% for sleep onset latency. Missingness did not differ between groups (*p* = 0.628). For the ESS, the interaction effect condition × time point was significant for the T0 versus T2 comparison (*β* = −1.05, SE = 0.43, *p* = 0.02), indicating a greater decrease in daytime sleepiness in the CBT‐I + light therapy condition (7.7–4.9) compared with the CBT‐I + placebo light condition (8.0–7.3). There was no significant condition × time point interaction effect for any of the other secondary outcomes, neither for the T0 versus T1 comparison nor for the T0 versus T2 comparison. With respect to saliva cortisol outcomes, the interaction effect condition × time point was not significant for the T0 versus T1 comparison (*β* = 75.81, SE = 105.96, *p* = 0.619).

## 4 Discussion

The present randomised controlled trial aimed at investigating the efficacy of light therapy as an add‐on to CBT‐I in a population suffering from insomnia disorder. The results showed no significant additional benefit of light therapy on insomnia severity, neither at post‐treatment nor at follow‐up. Regarding exploratory secondary outcomes, daytime sleepiness was lower in the light therapy (vs. placebo) condition at follow‐up.

In line with one of the few previous studies (Dekker et al. 2020), no additional significant effect of light therapy on insomnia severity was observed. Several reasons may account for this finding. First, the light intervention was administered at a low dose in the present study (i.e., four times per week for 20 min) to ensure feasibility for employed participants within their work schedules. Second, light therapy was applied for a relatively short period (31.3 ± 9.0 days; range 15–57 days). Thus, it is conceivable that the duration of the intervention was insufficient to generate significant effects on subjective sleep. Third, the ISI may lack sensitivity to detect subtle or short‐term sleep‐related changes due to the restricted sensitivity inherent in the 5‐point Likert scale. Moreover, light therapy and CBT‐I may engage overlapping mechanisms, including sleep regularisation (Maurer et al. 2018), which may have been more pronounced than originally anticipated and could have obscured effects attributable specifically to light therapy.

Regarding secondary exploratory outcomes, CBT‐I + light therapy had a significant effect on daytime sleepiness. This is line with findings of wakefulness‐promoting effects of light exposure, especially for short‐wavelength light (for a review, see Blume and Münch 2025). Interestingly, this effect became evident only at follow‐up. This is in line with previous findings reporting effects of light therapy primarily at follow‐up (Dekker et al. 2020; Leerssen et al. 2020). One possible explanation for these late emerging changes may be that light therapy may exert its effect primarily via a gradual phase shift in circadian timing (Zhao et al. 2025), requiring an extended time period to develop measurable effects on subjective sleep‐related parameters. Furthermore, this result gains additional relevance given that nearly half of the patients with insomnia consult a physician because of daytime impairments (Morin et al. 2006). However, sleepiness is typically not a primary focus of CBT‐I and meta‐analytic findings indicate small effect sizes regarding this symptom (Benz et al. 2020). Notably, an improvement of two to three points on the Epworth Sleepiness Scale has been suggested as the minimum clinically important difference in patients with sleep apnoea (Patel et al. 2018), providing a benchmark for clinically meaningful change in daytime sleepiness. Given its clinical relevance, it may be worth considering a stronger focus on daytime sleepiness as a treatment target for which the use of additional therapy tools—such as light therapy—could be beneficial.

Concerning sleep diary‐based sleep parameters, subjective sleep onset latency was reduced by 27.6 min (from 41.1 min at T0 to 13.5 min at T1) in the CBT‐I + light therapy group. However, this effect failed to reach statistical significance in comparison to the CBT‐I + placebo light condition (from 39.1 min at T0 to 24.8 min at T1). No follow‐up data were collected for this measure; thus, potential delayed effects on sleep onset latency, as reported by Dekker et al. (2020), could not be detected and should be addressed in future studies. The same applies to depressive symptoms; prior research on combining CBT‐I with chronobiological treatments, including light therapy, found mood‐stabilising effects only at 1‐year follow‐up (Leerssen et al. 2020). Such findings underline the need for study designs with extended follow‐up periods. Regarding the remaining secondary outcomes (including cortisol saliva samples, questionnaire‐ and sleep diary‐based outcomes), no significant results were found.

Several methodological strengths of the present study should be addressed. First, one major strength is the placebo control condition, which varied only in light characteristics while keeping the application modality identical across groups. This is in contrast to earlier studies in which placebo conditions differed largely from active conditions (e.g., Dekker et al. 2015; Leerssen et al. 2022). Second, as mentioned above, the light therapy intervention was not very time‐consuming, increasing its feasibility within working schedules. Third, the dropout rate was very low (i.e., only one participant). Nonetheless, limitations of this trial should also be addressed, including a relatively small sample size (i.e., small effects may not have been detectable). In addition, the treatment duration was not entirely standardised, which may have increased variability. Finally, light exposure during the treatment period was not systematically assessed. Therefore, interindividual differences in habitual and seasonal light exposure may have obscured the potential effects of the light therapy.

In conclusion, the present results do not demonstrate a clear direct effect of adjunct light therapy on insomnia severity. However, positive effects are evident with regard to daytime sleepiness at follow‐up. This indicates the need for future studies with larger sample sizes and longer follow‐up periods (i.e., up to 1 year) with a stronger focus on the improvement of daytime sleepiness.

## Author Contributions

**Sarah R. Schmid:** writing – review and editing; writing original draft; methodology; investigation; visualization; conceptualisation; validation; funding acquisition, formal analysis; data curation; project administration. **Christine Blume:** writing – review and editing; methodology; funding acquisition. **Johanna Ell:** writing – review and editing; investigation. **Nikolas Konzen:** writing – review and editing; investigation. **Matthias Apweiler:** writing – review and editing; investigation. **Bernd Feige:** writing – review and editing; data curation. **Bernd L. Fiebich:** writing – review and editing; methodology; resources. **Julian E. Schiel:** writing – review and editing; investigations; formal analysis. **Katharina Domschke:** writing – review and editing; resources. **Kai Spiegelhalder:** writing – review and editing; writing original draft; visualization; conceptualisation; validation; funding acquisition, formal analysis; data curation; project administration; supervision, resources.

## Funding

The study was funded by EUCOR—The European Campus EVTZ. C.B. was funded by an Ambizione grant from the Swiss National Science Foundation (SNSF; Project No. 201742).

## Ethics Statement

The local ethics committee of the Medical Center—University of Freiburg approved the study protocol (Application no. EK‐Freiburg: 21‐1641).

## Consent

All patients provided written informed consent before inclusion in the study.

## Conflicts of Interest

Katharina Domschke is a member of the Neurotorium Editorial Board, Lundbeck Foundation and has received speaker's honouraria from Janssen‐Cilag GmbH. Christine Blume has had the following commercial interests related to sleep and/or light: honouraria for invited talks and workshops from F.A. Hoffmann‐La Roche AG, L'Oréal, Swissline Cosmetics, Ruby Hotels and Vattenfall. Christine Blume is an elected member of the Daylight Academy. The other authors declare no conflicts of interest.

## Data Availability

The data that support the findings of this study are available on request from the corresponding author. The data are not publicly available due to privacy or ethical restrictions.
